# Supportive Management of Mucositis and Metabolic Derangements in Head and Neck Cancer Patients

**DOI:** 10.3390/cancers7030862

**Published:** 2015-09-03

**Authors:** Marcelo Bonomi, Katharine Batt

**Affiliations:** Section of Hematology and Medical Oncology, Wake Forest School of Medicine, Medical Center Boulevard, Winston-Salem, NC 27157, USA; E-Mail: kbatt@wakehealth.edu

**Keywords:** mucositis, head and neck cancer, chemotherapy and radiation, pain, nutrition

## Abstract

Oral mucositis (OM) is among the most undesirable, painful, and expensive toxicities of cytotoxic cancer therapy, and is disheartening for patients and frustrating for caregivers. Accurate assessment of the incidence of OM has been elusive, but accumulating data suggests that reported OM frequency is significantly less than its actual occurrence. It has been suggested that over 90% of head and neck cancer (HNC) patients receiving radiotherapy (RT) with concurrent cisplatin experience severe OM with symptoms of extreme pain, mucosal ulceration and consequent limitations in swallowing and achieving adequate nutritional intake. This panoply of symptoms inevitably impacts a patients’ quality of life and their willingness to continue treatment. In spite of all the advances made in understanding the pathophysiology of OM, there is still no prophylactic therapy with proven efficacy. Strategies to limit the extent of OM and to manage its symptomatology include basic oral care, supportive medications, nutritional support and targeting aggressive treatments to high-risk patients. This review focuses on OM recognition, preventive measurements, and symptom-management strategies.

## 1. Introduction

### OM

Oral mucositis (OM) develops in approximately 90% of patients receiving RT to the upper aero-digestive tract [1,2], particularly those undergoing higher intensity radiation schedules (*i.e.*, hyper-fractionated or accelerated RT [3,4]) with concurrent cisplatin [5]. The prevalence of severe OM has been reported in 75%–90% of this patient population [6]. With the addition of targeted therapies to current head and neck cancer (HNC) anti-neoplastic treatment regimens, the risk of OM has increased. For example, cetuximab, when added to standard HNC chemo-radiation treatment (CRT) regimens, resulted in an increased prevalence of clinically significant mucositis when compared to CRT alone [7].

The relevance of OM extends from patient-related quality of life issues to provider-related concerns for timely treatment delivery and symptom minimization and finally, to broader health-system implications including costs inherent in the treatment of OM. For example, severe OM delays the subsequent chemotherapy cycle in 35% of patients; in 60% of patients, grade 3–4 mucositis causes dose-reduction and in an additional 30% of patients, chemotherapy is discontinued altogether [8]. These complications can therefore, compromise the timely delivery of treatment with resultant impact on local and distant recurrences. From the provider perspective, severe OM compromising the oropharynx, larynx, and hypopharynx is extremely difficult to evaluate. Patients present with pain and dysphagia but the severity of inflammation, swallowing and mucosal compromise prevent direct assessment of the OM. The clinician is dependent therefore, on his/her best assessment based on patient symptom report. Given that 50% of patients can experience discomfort of the oropharynx and oral cavity for a prolonged period of time, even following resolution of visible OM [9], there are also long-term patient-related implications to OM [10,11]. It is estimated that the median cost of medical resources per HNC patient undergoing CRT reaches $39,000; those patients without mucositis require ~$21,000 medical resources, accounting for a median incremental cost to treat each HNC patient with OM of $18,000 per patient [12]. These costs are substantial and derive from hospitalization days, diagnostic tests, opioid use, and additional supportive measures, particularly an increased need for fluids and nutritional supplementation. Furthermore, the greater the severity of OM, the greater the associated costs of treatment.

## 2. Clinical Course and Impact of OM

The severity of OM ranges from superficial erythema with accompanying soreness, to full-thickness mucosal ulcerations and significant pain. These signs and symptoms are thought to correlate with the high levels of pro-inflammatory cytokines released during the development of OM [13,14]. Among patients being treated for HNC, mucositis follows a predictable and well-documented course [15]. Of patients receiving myeloablative chemotherapy, RT, or both to the head and neck area, OM is the most commonly cited bothersome adverse event associated with the treatment. By the end of the first week of treatment with typical cumulative radiation doses of 10 Gy, patients evidence erythema of the oral mucosa and complain of a burning discomfort similar in intensity to a food burn. This relatively mild pain continues to escalate between the second and third week of treatment (with cumulative radiation doses of 20–30 Gy) as frank mucosal ulceration develops. Lesions at this stage often necessitate a modification in food intake and a marked increase in the need for analgesics. As radiation progresses, individual ulcers frequently coalesce, resulting in a confluent injury affecting many aspects of the oral mucosa. Pain intensifies and is often so severe that even with aggressive therapy, it can be very difficult to control [16]. With the abrupt appearance of severe, extensive ulcers and/or ulceration affecting the keratinized mucosa of the dorsal tongue, gingiva or hard palate, there is also an increased association with infection [15,17].

It is clear then, that the clinical impact of OM is profound. Even when described as mild to moderate, OM is still associated with increased oral pain, weight loss, dietary modifications (including gastrostomy tube placement and subsequent use), dehydration, and reduced performance status [16]; even mild mucositis results in more frequent hospitalization and breaks in treatment [18]. Severe OM is associated with systemic findings of weight loss out of proportion to calorie intake, as well as fatigue, anorexia, dehydration, and general debility from CRT. Ulcerations, in addition to causing severe pain, are also a frequent site of secondary infections. In myelosuppressed patients, an ulcerated area can act as a portal for systemic bacterial spread [19]. Severe OM therefore, is strongly associated with an increased risk of bacteremia and sepsis [20]. In patients who develop ulceration in high dose areas or in the posterior regions of the mucosa, symptoms may persist for several months beyond expected healing, causing prolonged symptomatology [21].

OM is associated with a wide range of symptoms, a diminished quality of life (QOL) [6] and significant health and economic outcomes—increased analgesic and antibiotic use, increased number of febrile days, prolonged lengths of hospitalization, needs for parenteral nutrition, increased infectious risks—all of which result in increased resource requirements and healthcare costs.

## 3. Assessment of Mucositis

It is critically important to develop and validate methods that can quantify the degree of OM experienced by patients. This will allow the formulation of targeted interventions that efficiently combat this adverse chemotherapy-related outcome. The Multinational Association of Supportive Care in Cancer and the International Society for Oral Oncology (MASCC/ISOO) proposed that an OM assessment tool should be objective, sensitive, validated, reliable and easy to use in all clinical applications. An emerging body of evidence suggests that assessments of OM should include a standardized instrument or combination of instruments measuring not only physical and functional factors, but also patient-perceptions [14,22]. The World Health Organization (WHO) and the National Cancer Institute Common Terminology Criteria for Adverse Events (CTCAE) scales are the most commonly used, yet neither tool assesses OM incorporating all the proposed criteria [8]. The WHO scale is easy to use but is based only on clinical observations of erythema and ulceration paired with a patient’s ability to maintain an oral diet [15]. The National Cancer Institute Common Terminology Criteria for Adverse Events (CTCAE) scale is frequently used in clinical trials to document the side effects caused by different anti-cancer therapies. Of note, CTCAE v4.0 evaluates OM based on patient-reported variables such as pain, dysphagia and eating behavior, [23] thereby incorporating changes in QOL experienced while receiving CRT [24]. Overall, available assessment tools still concentrate on clinician interpretation, despite evidence that patient-reported symptoms tend to be more severe than those recorded by physicians. The OM Daily Questionnaire (OMDQ) however, was created to record patient-reported OM outcomes on a daily basis without requiring clinic visits. This questionnaire is designed to enable clinicians to more rapidly identify OM and therefore, implement early treatment and monitor subsequent clinical changes. In patients with laryngeal, oropharyngeal and/or hypopharyngeal cancers suffering from OM, direct visualization of the injured mucosa is extremely difficult, due to swelling, inflammation and pain preventing passage of a laryngoscope. Therefore, clinician and patient assessment exists as the only measure of treatment effectiveness [6].

## 4. Mucositis Prevention

A number of diverse interventions have been tested for the prevention of mucositis in HNC patients receiving CRT. Although many of these interventions (*i.e.*, “magic mouthwash”, chlorhexidine oral rinse) are available over the counter, used off-label or marketed as devices, the U.S. Food and Drug Administration (FDA) has approved none of these interventions for the prevention of OM. The MASCC/ISOO recently updated its clinical practice guidelines for mucositis prevention [25] to reflect the results of a systematic review reporting the beneficial effects of oral care protocols (combination of toothbrushing, flossing, and ≥1 mouth rinses to maintain oral hygiene) in the prevention of OM [25]. Although the data was not strong enough to support a full recommendation, there was enough positive evidence to favorably support the use of oral care protocols in preventing OM across all cancer treatment modalities. The same data also supported a suggestion against the use of chlorhexidine mouthwash for the prevention of OM in patients receiving RT for HNC [25].

Palifermin (keratinocyte growth factor-1) is the only agent approved by the U.S. FDA and the European Medicines Agency (EMA) for OM. This approval was based on a body of evidence that included a large, well-designed, randomized controlled trial as well as other supporting studies recommending the use of palifermin to prevent OM in patients diagnosed with a hematologic malignancy planned for high-dose chemotherapy, total body irradiation followed by autologous stem cell transplantation [26]. In the HNC patient population, two double-blind, randomized, placebo-controlled trials assessed palifermin’s efficacy in preventing OM [27,28]. Both trials found that the physician-assessed benefit of palifermin was not paralleled in the patient-reported outcomes (PRO) (assessed through the mouth and throat soreness score). Adverse events reported by physicians were less accurate than those reported by PRO instruments. This has led to a suggestion that clinician-assessment tools be replaced by PROs. While there is likely utility in both measures, enhancing an understanding of a patient’s symptoms through a combined reported outcome [29], the resultant findings from these studies do not support the use of palifermin in preventing OM in HNC patients.

Another potential agent considered in the prevention of OM is that of the granulocyte-macrophage–colony-stimulating factor (GM–CSF). A review of the evidence not only suggests against the use of granulocyte-macrophage–colony-stimulating factor mouthwash, but it also finds reduced local tumor control when used during HNC RT. In a randomized, multicentric trial evaluating the preventive effect of granulocyte colony-stimulating factor (G-CSF) in patients receiving hyperfractionated RT or concomitant CRT, there was an unexpected increase in locoregional failures experienced by patients with stage III–IV squamous cell carcinoma of the head and neck area [30].

Benzydamine hydrochloride is a nonsteroidal anti-inflammatory drug that can inhibit the production of proinflammatory cytokines such as tumor necrosis factor-α and interleukin-1b. The MASCC/ISOO mucositis guidelines recommends the use of benzydamine mouthwash to prevent OM in patients with HNC who are receiving moderate-dose RT (up to 50 Gys) without concomitant chemotherapy. In the initial study, prophylactic use of benzydamine was assessed with patients undergoing a weekly clinical evaluation before, during and up to a maximum of 3 weeks following the completion of treatment. The benzydamine group demonstrated a 30% reduction in the incidence of erythema and radiation-induced ulcers. In addition, 33% of patients who received the study drug did not show any signs of radiation-induced ulceration as compared to 18% of patients who received placebo (*p* = 0.037). Patients receiving benzydamine rinses experienced a significant delay in the need for opioid analgesia as compared to the group on placebo drug (*p* < 0.05) [31]. It is important to note however, that benzydamine did not show any clinical benefit in the group of patients that underwent a full course of accelerated RT [31]. In a second study evaluating the preventive effect of benzydamine oral rinses on the incidence of OM, subjects were again randomized to receive either the benzydamine rinses or a placebo drug. Unlike the previously described study, patients receiving a definitive course of RT or concomitant chemo-radiotherapy (CRT) for HNC were included in the study. Data from this study demonstrated that patients using the benzydamine rinses had only a 44% incidence of severe OM in comparison to the placebo group with a 79% incidence rate (*p* = 0.001) [32] of severe OM. In conclusion, both trials support the clinical utility in prophylactic use of benzydamine oral rinses with moderate doses of RT. Whether this conclusion can be broadened to patients receiving concomitant CRT is still unclear as the included sample size in both trials is too small from which to derive meaningful conclusions [31,32].

Low-level laser therapy (LLLT) is a local application of a monochromatic, narrow-band, coherent light source. The MASCC/ISOO guidelines suggest that low-level laser therapy (wavelength around 632.8 nm) can be used to prevent oral mucositis in patients undergoing radiotherapy, without concomitant chemotherapy, for head and neck cancer (Level of evidence III). [33] The mechanism of action of LLLT remains unclear; it seems to have a protective effect at the cellular level during periods of oxidative stress in patients receiving head and neck RT [34]. In a recent meta-analysis evaluating the preventive effect of LLLT, a reduction in the incidence of severe OM was observed. An even larger clinical benefit was detected when the analysis was restricted to trials including patients who received LLLT doses greater than 1 Joule [35]. In a double-blind, randomized trial comparing two LLLT groups receiving daily LLLT of different dosages throughout the full course of RT (Group 1 LLLT dose: (660 nm/15 mW/3.8 J/cm^2^/spot size 4 mm^2^; Group 2 LLLT dose: 660 nm/5 mW/1.3 J/cm^2^/spot size 4 mm^2^), patients randomized to the high-dose LLLT (group 1) had less severe OM and reduced pain during RT [36]. It is important to note that there are inconsistencies in the reported clinical benefits of LLLT given the lack of standardized laser parameters and the differences in described dose levels reported in the literature. Beyond these inconsistencies, no safety issues have been reported to date in the long-term use of LLLT [36]; however, long-term data is limited and longer follow-up periods are needed to definitively ascertain the full spectrum of possible safety issues.

## 5. Mucositis—Associated Pain

OM pain is reported in the majority of HNC patients undergoing treatment [8]. It interferes with daily activities of living in approximately one-third of patients and with social activities and mood in 50%–60% of patients [34]. Pain usually arises from the activation of primary nociceptive afferents by tissue-damaging stimuli and the subsequent processing of this activity within the nociceptive system. It can also be generated within the nociceptive system either initiated by a primary lesion or from overall dysfunction of the nervous system [37]. This type of pain is termed neuropathic pain. In a prospective study of patients with upper aero-digestive tract tumors, neuropathic pain was diagnosed in 56% of the studied subjects, although the primary pain presentation was a mixed picture [38]. As previously discussed, there is an increased frequency of patients with severe OM developing local infections, typically from Candida sp and Herpes virus family organisms [17,39,40]. Patients with local infections usually evidence higher pain and dysphagia complaints and often describe a “neuropathic” component during the pain episodes. The correlation between pain severity and infectious potential is important to recognize, as it implies not only the use of local anesthetic and analgesic medications, but appropriate antimycotics, antivirals and/or antibiotics. This subset of patients may evidence a pain severity that necessitates dose-escalating systemic analgesics in addition to local measures [39,41].

## 6. Opioid Use for Management of Mucositis—Associated Pain

Morphine is the classic example of an opioid used for its analgesic effects. These effects result from its capacity to bind to opioid receptors in the central nervous system as well as those in the periphery. Basic science research suggests that morphine provides analgesic relief when locally applied to tissues damaged from a series of inflammatory events [42,43,44]; this has also been observed in clinical scenarios. In HNC pain, the topical use of morphine has further relevance. Firstly, morphine preferentially binds µ-receptors with local application [45]. Secondly, morphine has poor transmucosal and sublingual absorption. The absorption capacity of a given drug depends on the pK of the drug, the pH of the absorbing tissue and on the drug’s lipid solubility [46,47,48]. Morphine is poorly lipid soluble and is optimally metabolized in a low pH. The oral cavity of a patient suffering from OM represents the ideal conditions for morphine with inflamed tissues creating direct receptor exposure in a low pH setting. In a study performed on HNC patients with primary tumors of the nasopharynx, oropharynx and oral cavity treated with concomitant CRT, twenty-six patients were randomized to morphine mouthwashes, or a formulation of “magic mouthwash” comprised of a mixture of magnesium aluminum hydroxide, viscous lidocaine and Benadryl. In the morphine arm, patients were instructed to use an oral rinse of 15 mL 2‰ morphine solution (2000 mg morphine diluted in 1000 mL of water) on an as needed basis every three hours, to hold the mouthwash for 2 min and to avoid swallowing the oral rinse. The “magic mouthwash” group was given the same instructions. In an analysis of the two groups, severe pain was experienced for, on average, 3.5 days less in the morphine group than in the group using “magic mouthwash”. In addition, the majority of patients randomized to the “magic mouthwash” group also needed systemic opioids for pain control [49]. In another two-block pilot design study looking at patients with loco-regionally advanced upper aero-digestive tract tumors receiving definitive concomitant CRT, different morphine dosages and pK analyses of the solutions were performed. The patients were randomized to receive two differently dosed morphine oral rinses. Group 1 used 15 mL of 1‰ morphine solution; Group 2 used a 2‰ diluted morphine solution. In the extended phase (block 2), twenty-two patients were included to assess the efficacy and safety of the morphine solution diluted at 2‰. As expected, the group of patients receiving the higher dosed oral morphine solution (2‰) had better pain control than the group of patients receiving the lower dosed morphine solution (1‰). Interestingly, pK studies did not show any clinically significant differences between the two morphine concentrations [39].

Much of the evidence used to manage OM-associated pain is extrapolated from the bone marrow transplant literature. Another well-described systemic opioid with marked efficacy in this setting is fentanyl, a short-acting synthetic opioid with potent analgesic capabilities that can be administered intravenously or subcutaneously. The low molecular weight, high potency, and high lipid solubility of fentanyl allows it to be delivered easily transdermally where a controlled-release membrane allows for a 72-hour reservoir to be steadily absorbed through the skin into the microcirculation and subsequently, systemically into the bloodstream. The variety of available transdermal dosages allows for easy titration, particularly in patients with compromised swallowing facilities. Two prospective trials have shown that the routine use of transdermal fentanyl reduced the intensity of pain and improved the quality of life of patients suffering from severe OM [50,51].

Breakthrough pain is a common occurrence in HNC patients, particularly during swallowing, coughing (expulsion of mucus) and at otherwise unforeseen moments. Difficulties in swallowing can limit the efficacy of traditional pills; PEG-tubes, while offering a secondary route for medication delivery, are compromised in that they cannot often accommodate opioid tablet sizes. Crushing opioids usually impacts the efficacy of the drug. At present, breakthrough pain has relied on liquid formulations of opioids—a delivery mechanism that can accommodate patients with a degree of swallowing ability as well as those with PEG tubes.

Innovation around pain management has focused predominantly on mechanisms of prognosticating mucositis severity in the HNC patient initiating therapy. While still in early phase development in HNC patients, the technique of modeling symptom clusters through the combined use of computational biology technologies with molecular, clinical and genetic tissue markers is a means of risk stratifying mucositis severity. This, in turn, will help early and effective identification of patients at risk [52].

## 7. Adjuvant Analgesic Therapies

In patients with OM-associated neuropathic pain or a mixed pain picture, there is a definite role for adjuvant therapies [53]. Two retrospective studies that evaluated the addition of gabapentin (median dosages of 2700 mg/day) to OM-associated pain regimens in HNC patients treated with RT or concurrent CRT, suggested a potential decrease in opioid for adequate pain control [54,55]. In a randomized trial evaluating opioids *versus* tricyclic antidepressants for OM-associated pain in HNC receiving RT, up to 40% of patients achieved sufficient pain control on tricyclic antidepressants alone [56]. Although no randomized trials to date have evaluated the use of anticonvulsants and anti-depressants with opioids for the management of OM pain with neuropathic features, the available data suggests that the combined use of these medications may offer additional pain control.

Doxepin hydrochloride is a tricyclic antidepressant that has demonstrated anesthetic and analgesic properties with topical administration. In a recent randomized trial organized by the Alliance Cooperative Group, oral rinses with Doxepin diminished the intensity of OM-associated pain as compared to placebo. However, no reduction in the opioid dosing was observed in this study [57]. This suggests that further studies are warranted to determine the role of doxepin oral rinses in the management of OM.

## 8. Nutrition, Hydration and Electrolyte Support during Treatment

The maintenance of adequate nutritional support is imperative in patients with malignancies of the upper aero-digestive tract for whom the goal is definitive CRT with curative intent. Without these supportive measures, the data evidences an increased risk of treatment interruptions resulting in a reduction in tumor control and overall survival [58]. Two primary measures have shown an impact on achieving nutritional goals: (1) regular dietary counseling during CRT [59] and (2) an emphasis on protein-related goals. Dietary counseling seems to not only maintain nutritional status, but potentially improve it [59]; patients who maintain good protein stores evidence less severe OM [60]. Yet patients undergoing definitive CRT with varying degrees of OM will have swallowing difficulties and therefore, will struggle to stay abreast of nutritional requirements. To date, recommendations for prophylactic enteral feeding include patients with a pre-treatment body mass index (BMI) less than 20, large primary tumors and/or with hypopharyngeal involvement, and/or with dysphagia prior to the start of treatment [61]. While nearly all patients require some oral nutritional supplementation during treatment, 50%–70% of patients treated with CRT will have severely impaired swallowing and require an enteral feeding tube during or immediately after treatment, usually with a percutaneous endoscopic gastrostomy tube (PEG) or a nasogastric tube (NGT) [61,62]. The choice of PEG tube feeding *versus* NGT feeding [61,62] has implicit cost and quality of life implications, yet the data suggests that the increased expense and duration of PEG tube feeding have significant benefits in terms of patient outcomes. Whether placing a prophylactic feeding tube is preferable to a more reactive approach is still a matter of controversy. Several centers routinely place prophylactic feeding tubes, generally PEG tubes, before beginning definitive CRT in HNC patients. This issue was addressed in a randomized trial of patients with advanced HNC, finding prophylactic PEG tube use was associated with a significantly earlier start and longer use of enteral nutrition but with fewer malnourished patients and an improved health-related quality of life (HRQoL) at 6 months post-treatment. Of particular note, the group randomized to prophylactic PEG tube placement had higher ratings of physical function, role function, and cognitive function as well as significantly less fatigue and feelings of illness [63].

Poor nutritional status also translates to potential electrolyte abnormalities. A review of lab values in patients undergoing concomitant CRT not maintaining adequate nutritional support, suggests a significant relationship between creatinine percent or BUN rise, and percent body weight loss. Dehydration associated with severe dysphagia during CRT and an inability to feel thirst is the likely causative actor [64]. In our experience, the routine addition of daily parenteral hydration during the last two weeks of radiotherapy is associated with improved renal function, less fatigue and better outcomes. This again highlights the importance of routine nutritional counseling, the prophylactic use of PEG tubes, and the implementation of hydration methods above and beyond standard hydration for patients with advanced HNC receiving CRT, particularly cisplatin.

Re-feeding syndrome is an under-recognized side effect of CRT treatments. Usually defined as a potentially fatal syndrome in which dangerous fluid and electrolyte shifts occur in malnourished patients undergoing artificial re-feeding by enteral or parenteral routes, it is a complex syndrome with hypophosphatemia as the hallmark feature. Other electrolyte imbalances that can occur include changes in glucose, protein and fat metabolism, abnormalities in sodium and fluid balance as well as thiamine deficiency, hypokalemia and hypomagnesemia [65]. The main cause of re-feeding syndrome is rapid re-feeding following a period of undernourishment; the mode of feeding is not important. As a result of the metabolic changes in early starvation the body switches the main energy source from carbohydrate to protein and fat. The basal metabolic rate decreases by as much as 20%–25%. As fasting continues, the body aims to conserve muscle and protein. Therefore, in an attempt to conserve ketone bodies, tissues switch to fatty acids for their energy source. An increase in blood levels of ketone bodies ensues, thereby stimulating the brain to convert to using ketone bodies as its main energy source. As a result, the liver decreases its rate of gluconeogenesis, thereby conserving muscle protein. As a result of all of these metabolic changes, several intracellular minerals become severely depleted, even though the serum concentrations of these minerals (including phosphate) may remain normal or near normal. When feeding is restarted, the body metabolism suddenly changes from catabolism to anabolism. The re-introduction of carbohydrates stimulates insulin release, leading to the uptake of glucose, potassium, magnesium phosphorus, and water into cells. Furthermore protein and fat synthesis are stimulated, further consuming minerals. As cells continue to be produced, available mineral reserves are consumed. These changes result in intra- and extracellular mineral deficits, resulting in the clinical complications of re-feeding syndrome [63]. The importance of this problem was demonstrated in a survey of HNC patients receiving RT or CRT in which approximately 14% of patients were admitted with severe malnutrition and risk of re-feeding syndrome [66]. 

To effectively prevent re-feeding syndrome, a thorough nutritional assessment needs to be carried out before re-feeding is initiated. Important factors to assess include weight loss over time, current nutrition, existing social issues including alcohol intake, and any underlying psychological issues. Baseline plasma electrolytes, especially phosphate, sodium, potassium and magnesium, and glucose should be measured before feeding and closely monitored and corrected during re-feeding. For patients at high risk of developing re-feeding syndrome, repletion of calories should be started slowly (maximum 10 kcal/kg/24 h) with a planned slow increase to meet individual patient needs over 4–7 days. In patients who are very malnourished (e.g., BMI less than 14 kg/m^2^ or negligible intake for 2 weeks or more), the NICE guidelines recommend re-feeding start at a maximum of 5 kcal/kg/24 h in combination with cardiac monitoring, due to the risk of cardiac arrhythmias [67]. In general, re-feeding should introduced at no more than 50% of caloric requirements in patients who have eaten little or nothing for more than 5 days. The rate can then be increased if no re-feeding complications are detected on clinical and biochemical monitoring [67]. Potassium, phosphate and magnesium require continuous assessment with immediate correction to avoid deleterious effects, particularly cardiac events [67].

In summary, re-feeding syndrome, while easy to prevent and detect, is often overlooked. Patients with HNC are a patient population with multiple risk factors for nutritional depletion including malignancy-associated cachexia, dysphagia and odynophagia, iatrogenic treatment effects and pre-existing detrimental social behaviors and therefore, are at increased risk of re-feeding syndrome.

## 9. Conclusions and Future Directions

As our understanding of the pathophysiology of OM begins to include underlying molecular mechanisms, new potential targets for therapeutic interventions will be identified. To date, different topical and systemic medications are being used, yet no single efficacious intervention or prophylactic agent has emerged as the leading treatment in the management of CRT-induced OM. Although several ongoing clinical trials are looking into manipulating the inflammatory cascade associated with CRT-induced tissue damage, standard preventive techniques are still focused on adequate oral hygiene, pain management with topical and systemic analgesics and timely identification of symptomatic complaints that herald an infectious process. Supportive measures that address nutritional intake, electrolyte management, dehydration and aggressive pain management are still, the best means of preventing OM.
